# Foreign Summary

**Published:** 1839-02-16

**Authors:** 


### FOREIGN SUMMARY.

*Case of extensive Detachment of the Skin.* By the late JAMES WIER, M. D.—C. Cowans, aged 11, a stout, healthy, and active girl. On the forenoon of October 4, 1825, while handing sheaves of barley to her father, who was feeding a threshing-mill newly put up, she inadvertently approached so near to the machinery, which was still uncovered, that her hair, which was coiled up behind, was caught by the teeth of the pinions which turned the rollers, and dragged inwards. Before she could be rescued from her perilous situation, the integuments had been extensively detached from the upper part of the head and the posterior part of the neck. The skin had burst over the superciliary ridges, stripping off the cilium of the left eye, detaching almost the whole of the cartilage of the left ear, leaving it hanging by its lobe, and exposing the muscles covering the angle of the jaw; on the right side, it had given way immediately above the cilium, and was detached downwards, forming a curve around the right ear, leaving it almost insulated. The separation of the skin proceeded downwards, denuded all the posterior part of the neck, exposed part of the muscles of the left scapula, and reached to the edge of the right; below this it became more narrow, assuming the shape of the *trapezii* muscles, and when the father, by an Herculean exertion of strength, had stopt the machinery by laying hold of the spur wheel, it had extended nearly to the last dorsal vertebra. By turning back the machinery, the detached scalp and skin were unravelled, and were scarcely lacerated from the manner in which the rollers were constructed to take in the sheaves, except a small part where the scalp was first laid hold of. As they proceeded to convey her to the house, the father, seeing the detached skin trailing behind, took out his knife, and unfortunately cut it off as far down as the last cervical vertebra.

Being from home when the messenger despatched to request my presence arrived, my assistant, Mr. J. Marshall, set off with all possible speed, and found matters in the state above described. Two hours might intervene between the time of the occurrence of the accident and the arrival of my assistant, the distance being six miles. On the cause of the deficiency of the integuments being explained to Mr. Marshall, though the hopes of procuring any adhesion with the portion removed, and the parts from which it had been stripped, were certainly very improbable, yet, as no harm could accrue from the attempt, he cleared it of the hair and beards of barley, and carefully secured it in its place by stitches and adhesive plaster.

I saw her next day, and found her pulse about 120, and feeble, though, from the nature of the accident, she had lost but a very few ounces of blood. Skin nearly of the natural temperature. Some wine was directed to be given.

8th. On removing the dressings, we perceived that not only that portion of the skin which had been detached by the knife, but also three or four inches of the upper part of that which had been left adherent, had lost their vitality, and resembled a slough about to be thrown off. The exposed surface was dressed with simple ointment, and the wine was continued along with a farinaceous diet.

10th. The greater part of the remaining dead skin was washed off, and healthy granulations had appeared on the cranial surface. The pulse was still feeble and frequent. She complained very little of pain. The wine was continued.

12th. I was much disappointed, upon removing the dressing to observe that the granulations had disappeared. Her pulse could scarcely be counted, and was very feeble. The wine was increased. A cupful of beef-tea daily was ordered.

14th. Some slight appearance of the return of the granulations upon cranial surface. The pulse was firmer, still very feeble. The wine and beef-tea were continued.

16th. Granulations numerous on the cranial surface, though scarcely apparent on the other parts of the sore. The discharge of purulent matter was pretty copious. No appearance of union betwixt that portion of the skin which still retained its vitality and the subjacent surface.

18th. Granulations had again disappeared, and the discharge of purulent matter was diminished. Pulse again more feeble, and she was altogether worse. The left external ear still un-united. Six ounces of wine and a pound of beef-tea daily were ordered to be given.

20th. Granulations beginning to show themselves on the cranial surface. The pulse was stronger and less frequent.

22d. Granulations more apparent upon cranial surface, and also beginning to present themselves on the other parts of the sore. Pulse improving in strength.

28th. Appearance of sore continuing to improve. Severe inflammation of left eye.

From this time, she progressively gained strength, and the surface of the sore retained its healthy appearance. The left external ear, which had been frequently replaced, was neglected in my absence for a few days, and was found adhering to the neck where it was allowed to remain. She soon became able to sit up in bed, reclining her head on her knees. New skin began to shoot from the edges of the old. The palpebræ of the left eye were drawn towards the ear, and the opening between them extended to nearly three inches in length, leaving the eye permanently exposed. The constant inflammation of the eye which this produced, terminated at last in staphyloma. Two months after the accident, her strength was so much improved, that she began to employ herself in sewing.

During the following summer, she was able to go out to the open air when the weather was favourable. That portion of the skin which retained its vitality had now occupied its former place, but the new skin extended very tardily from the edge of the old, more particularly on the cranial surface. No insulated portions of skin ever appeared on the surface of the sore.

About three years and three months from the time of the accident, she became affected with gangrene of the left foot, apparently from neglected chilblains. The gangrene continued to extend towards the trunk, and she died in the course of ten days. By the approximation of the old, and the production of new integuments, the neck was skinned over as high as the third cervical vertebra, but the granulations on the upper part of the neck, the occiput, the *ossa parietalia*, and part of the squamous portions of the temporal bones, still remained uncovered at her death. She had grown taller, but never recovered her former plumpness.—*Ed. Med. and Sur. Journal, Oct.*, 1838.

---

*Aneurism at the bend of the arm consequent upon venesection—operation.*—P. N., aged 17, from Milnathort, was admitted on the 9th of August, on account of a tumour which had occurred at the bend of the right arm, in consequence of a bleeding performed two weeks previously for some affection of the throat. Nothing, he says, happened at the time to occasion alarm, but two hours afterwards, the arm swelled, so as to excite his apprehension, which was relieved by the operator's assurance that there was nothing that would not soon be dispelled by friction with some stimulating liniment. General swelling of the arm nevertheless took place, and when this subsided, a diffused pulsating tumour was found at the bend of the arm, above and below which it extended for nearly three inches. The arm could not be extended beyond a right angle, and there was hardly any pulse to be felt at the wrist. After trying compression for a few days, without any benefit, I took the advice of my colleagues in regard to the case, and was by them advised not to delay the operation any longer, as three weeks had elapsed, and the state of matters was not improving.

I therefore, after applying a tourniquet to the arm, made a free incision into the aneurism, turned out the clots, and tied the artery above and below the wound, which was nearly a quarter of an inch in length. In a former report I have recorded a case of this kind, in which I tied the brachial artery without opening the sac, but was afterwards obliged to do so, from the disease not being controlled by this measure.—*Syme's Report of Surgical Cases, Edinburgh Medical and Surgical Journal.*

---

*Aneurism of the External Iliac Artery—obscurity delaying an operation—mortification of the limb—ligature of the common iliac, and amputation of the thigh—dissection.*—On the 15th of May last I was requested by Dr. Alison, to see a case of iliac aneurism in a man who had come from the country. I found the patient, Alexander M'Dougal, aged 31, a thin, anxious, unhealthy looking person, by occupation a tailor. He stated that, three months before, he had perceived a beating tumour, the size of a pigeon's egg, in the right groin, which rapidly increased in size. A medical practitioner in the part of the country from which he came, prescribed fomentations, liniments, and poultices, with the view of hastening its progress to a proper state for being opened. As it gradually increased in size both upwards and downwards, without becoming softer or appearing to approach the surface, leeches and mercurial ointment were next employed, and no benefit being derived from these means, poultices of potatoes were recommended. He then applied to another practitioner, who told him that an operation would be required, and a third gentleman gave him the same opinion.

Having carefully examined the tumour, I came to the conclusion that it was an aneurism still within command by tying the common iliac artery, and advised him to go into the hospital. He did so, and in the case which I dictated next day to my clerk, it is stated, that "there is now a large tumour occupying the whole space between the pubes and the crest of the ilium, and extending three inches above a line drawn between these two points, and nearly two below it. The consistence of this tumour is tense and elastic; a very obscure pulsation may be felt at some parts of its extent; and the aneurismal *bruit* is very distinctly heard. The patient complains principally of pain in the knee."

My colleagues, Sir George Ballingall and Dr. Campbell, could not perceive any pulsation, but from the history of the case, and the general character of the tumour, agreed with me in regarding the tumour as an aneurism. With their their sanction I resolved to tie the common iliac artery, and desired the usual notice of an operation to be issued. In the evening, Dr. Alison, who at my request had again examined the tumour, sent me a note to say that he felt strongly inclined to think that it was not an aneurism, and I therefore resolved not to proceed without farther observation of the case. Next day a great number of medical gentleman assembled to see the operation which had been announced, and I took the opportunity of getting their opinions as to the nature of the tumour. Sir Charles Bell, Sir William Newbigging, Sir George Ballingall, Drs. Abercrombie, Davidson, Maclagan, Hay, Campbell, Robertson, &c. &c., carefully examined the swelling, and came to the unanimous conclusion that it would be wrong to operate until the nature of the case became more distinctly manifested.

After this, the tumour obviously enlarged, and acquired a more irregular and modulated surface,—the limb became œdematous. The pain in the knee, too, which had been very severe, seemed now quite intolerable, not only rendering the patient completely sleepless, but inducing him to sit constantly in bed with his head bent forward, almost in contact with his knees; and his complexion, which had been always sallow and unhealthy, assumed more of the greenish-yellow hue attending malignant disease of the cerebriform kind. Some persons, who at an earlier period had hastily and confidently decided upon the tumour possessing a solid consistence, then boasted of their superior acumen, and the patient seeing no prospect of relief, resolved to return home. As it was very desirable to see the issue of the case, and as it would have been impossible for the poor man to obtain at his own residence the means necessary for palliating his sufferings, I persuaded him, since he was resolved to leave the Infirmary, to go into the private hospital at Minto House, which he accordingly did on the 4th of June.

On the 6th the leg became cold, and on the 7th pulsation was felt distinctly throughout the tumour. On the 8th I requested Sir Charles Bell, Sir George Ballingall, and Dr. Campbell, to consider what should be done. The pulsation left no doubts as to the existence of aneurism. But the tumour had ascended to within an inch of the umbilicus. And the leg was not only cold, but of a bluish-purple colour from the knee downwards, with some large vesicles on the calf. It was plain that the mortification, if allowed to proceed, must prove fatal in two or three days at most. There was reason to think that room still remained for tying the common iliac; and that if this were done, the process of mortification might be stopped, by amputating the thigh. We therefore resolved that an attempt should still be made to save the patient's life.

The external incision was between six and seven inches long, extending from a little above the external ring upwards in the direction of Poupart's ligament, but diverging from it with a slight curve inwards. The parietes of the abdomen were readily divided, and little difficulty was experienced in turning back the peritoneum from the tumour, which was done cautiously to prevent rupture of the membrane on the one hand, or of the sac on the other. I felt the external iliac artery beating on the upper surface of the tumour, and traced it back until it became free, immediately beyond which the internal iliac came under the finger, and beyond this the common trunk lay quite free. So far the operation, however formidable, had not been attended with much embarrassment. But in proceeding to pass a ligature round the vessel, I found the narrow space, which was all that could be gained between the unyielding convex surface of the tumour, and the peritoneum distended by the viscera, and which was nearly equal in depth to the whole length of my hand, rendered the employment of aneurism needles very perplexing and uncertain. Having tried several of different kinds, I at length succeeded in passing one of the simplest form; and then, having the parietes of the cavity held carefully aside by iron spatulas, got a view of the ligature, and drew it out by means of a hook. A single firm knot was tied on the vessel, and one end of the thread cut away. The edges of the wound were stitched together.

In the course of the day the tumour became smaller and softer. The coldness and discolouration of the limb extended above the knee, and the patient complained of inability to retain anything in his stomach. On the 9th he was much in the same state, with some tympanitic distension of the abdomen. On the 10th he was no worse. On the 11th amputation of the thigh was performed close above the discoloured part of the limb. On the 12th the patient died.

On dissection, we found the common iliac firmly tied, exactly at the middle point between its origin and bifurcation, without any inclusion or injury of the neighbouring parts. The vessel contained a clot above and below the obstruction. The peritoneum showed traces of much inflammation, but not general or very extensive. The nodular inequalities of the surface of the tumour depended on the glands of the groin, which were enlarged and elevated by the subjacent swelling. The aneurism was of great extent, occupying the triangular hollow of the thigh, and stretching up into the pelvis, so as to fill the cavity of the *ilium*, and even extend considerably beyond it towards the back. The ramus of the pubes was exposed and rough, and the capsule of the joint was nearly, if not completely, perforated by absorption.

The external iliac, and its continuation, the common femoral artery, lay intimately incorporated with the aneurismal sac, but remained quite entire, except for about an inch at Poupart's ligament, where the coats of the vessel were deficient to this extent, on its inner or inferior surface.—*Ib.*

---

*Partus per Anum.*—Dr. Mekeln, of Kettwig, was called to a female on the 1st of January, who had given birth to a strong and living infant through the anus, two hours before his arrival. The wound in the under part of the vagina, as well as that in the rectum, was of great size. The perinæum, from the aperture of the anus to the vagina, was two-thirds torn, and very painful.

After three days, both the urine and fæces passed by their ordinary channels.

On the fourth day suppuration occurred; the wounds healed, and the woman in due course recovered her strength. Dr. Mekeln declares that he could discover no defect in the organization of the parts. The midwife states that at her arrival, she found the head of the child in the rectum.—*Dublin Journal, from the General Sanitäts-Berichte des Königlichen Medicinal-Collegium's.*

---

*Tinea Capitis.*—With the best success I have employed the ointment of Jasser in this complaint: its composition is

℞. Sulph. Purific. Vitriol Albi, āā ℥ij.—Axungiæ Porci recent. ℥vj.

M. ft. ung.

With this ointment let a portion of the head be rubbed,—after which, in some days, cracks in the scruff occur, and it then peels off. Every eight days I give a laxative of mercury, and prescribe a decoction of the woods, by which means this disease is generally cured in the space of from four to five weeks.—*Ibid, from Hufeland's Journal, 3d Part,* March, 1837.

